# Relationship between personality and sleep: a dual validation study combining empirical and big data-driven approaches

**DOI:** 10.3389/fpsyt.2025.1596269

**Published:** 2025-07-17

**Authors:** Lei Cao, Jiake Wu, Mengyao Wang, Liang Zhao, Xin Wang, Bowen Yao, Qi Li

**Affiliations:** ^1^ Beijing Key Laboratory of Applied Experimental Psychology, National Demonstration Center for Experimental Psychology Education (Beijing Normal University), Faculty of Psychology, Beijing Normal University, Beijing, China; ^2^ School of Information Management, Key Laboratory of Semantic, Publishing and Knowledge Service of the National Press and Publication Administration, Wuhan University, Wuhan, China; ^3^ The Institute of Biomedical Engineering, University of Oxford, Oxford, United Kingdom; ^4^ School of Economics and Management, Beijing Jiaotong University, Beijing, China

**Keywords:** personality, sleep, semantic understanding, neural network, microblog

## Abstract

**Objective:**

Sleep is a vital component of individual health, and personality traits are key factors influencing it. This study aims to investigate the relationship between personality traits and both modelassessed sleep problems and self-reported sleep quality.

**Methods:**

Using deep semantic understanding technology, we developed three deep learning models based on microblogs. Model 1 and Model 2 identified whether a post indicated a sleep problem, while Model 3 assessed the user’s personality traits based on the Five-Factor Model (FFM). We surveyed a dataset comprising 336 active users and then applied the models to a large-scale microblog dataset containing 4,860,000 posts from 15,251 users.

**Results:**

Our experimental results revealed that: (1) conscientiousness, agreeableness, and extraversion are associated with better sleep quality, while neuroticism is linked to poorer sleep quality; (2) the relationships between sleep problems and personality traits remained consistent when the model, trained on a small survey dataset with expert annotations, was applied to the large-scale dataset.

**Conclusions:**

These findings highlight the potential of using deep learning models to analyze the complex relationship between personality traits and sleep, offering valuable insights for future research and interventions.

## 1 Introduction

### 1.1 Background

Numerous studies have demonstrated that sleep plays a crucial role in physical recovery, memory consolidation, and emotional regulation (1). Scholars consider sleep to be a multifaceted phenomenon, proposing various sleep-related characteristics such as sleep quality (2), sleep duration (2), sleep problems (3) and sleep disorders (e.g., insomnia) (4). When these sleep issues arise, they are associated with an increased risk of developing various physical health conditions, including diabetes (5), obesity (6), cardiovascular diseases (7), and Alzheimer’s disease (8), as well as a heightened risk of mortality (9). In addition, sleep disorders are strongly linked to mental health problems, such as the experience of excessive negative emotions and insufficient positive emotions (10). To better understand sleep, researchers have developed various tools to assess different aspects of sleep, which can generally be categorized into objective and subjective measures. Objective sleep characteristics are typically measured using laboratory equipment or specialized wearable devices, to record multiple physiological signals during sleep (e.g., electroencephalography and eye movements). These measures are considered the standard clinical procedure for diagnosing sleep-related disorders. Subjective sleep characteristics are commonly assessed through self-reported questionnaires [e.g., the Pittsburgh Sleep Quality Index (PSQI) (11)] and sleep diaries.

Personality is considered one of the key factors related to sleep (4, 12). According to Akram et al. (4), the inherent nature of personality serves as both a predisposing and a potential maintaining factor for insomnia. A closer examination of personality’s susceptibility and its long-term effects can provide a better understanding of the etiology of insomnia (12). For example, Grandner (13) noted that elevated arousal levels may explain the connection between neuroticism and sleep quality, as neuroticism is associated with stronger physiological responses to stress, which can delay an individual’s return to a calm state after experiencing acute stress. To better understand personality, scholars have proposed various models from different perspectives. These include Cloninger’s psychobiological model (14), Cattell’s 16Factor Personality model (16 PF) (15), Eysenck’s personality model (16), the Alternative Model of Personality Disorders (AMPD) (17), and the Five-Factor Model (FFM) (18). Among the commonly used models, Cloninger’s psychobiological model emphasizes the integration of genetic and developmental factors and is widely applied in mental disorder research (14); the DSM-5’s Alternative Model of Personality Disorders (AMPD) focuses on traits such as negative affectivity, detachment, antagonism, disinhibition, and psychoticism, which are typically used to assess maladaptive personality traits and disorders (17). While all these models provide comprehensive analyses of personality, the FFM, also known as the Big-5 (19), stands out due to its demonstrated moderate-to-high longitudinal stability, reliability, and cross-cultural applicability (2). The FFM includes five broad traits: extraversion, agreeableness, conscientiousness, neuroticism, and openness to new experiences (18, 20).

Previous studies have shown that personality traits negatively affect various sleep characteristics, particularly sleep quality and chronotype (2, 3). In research based on the Five-Factor Model (FFM), neuroticism has consistently been identified as a stable negative predictor of sleep quality (2, 10, 21–25). Individuals with high neuroticism are more likely to hold metacognitive beliefs about sleep difficulties (24), experience more negative and fewer positive emotions (25, 26), and tend to be more hyperaroused, all of which adversely affect sleep quality (10). Beyond FFM-based research, the Cloninger model also shows a correlation between traits like harm avoidance and sleep disorders such as insomnia (27). Zakiei et al. (28) emphasized that not only traditional personality traits but also pathological traits (e.g., psychoticism and negative affectivity) are stable predictors of various sleep problems. Akram et al. (4) also found that insomnia is linked to negative or maladaptive personality traits, including neuroticism, perfectionism, worry, social inhibition, and avoidance. Hisler et al. (29) suggested that trait stress, rather than neuroticism, modulates the relationship between sleep and self-control. However, several recent studies have reported findings that challenge these widely accepted relationships, particularly the link between neuroticism and sleep quality (25, 30). Saksvik-Lehouillier et al. (25) found that individuals with high neuroticism experience fewer negative emotions under partial sleep deprivation than during normal sleep, whereas those with medium and low levels of neuroticism reported the opposite pattern. A further study of college students using Actigraph to objectively measure sleep found no relationship between neuroticism and sleep quality (30).

Several studies suggest that conscientiousness and extraversion are positively correlated with sleep quality (2, 21, 23), as individuals higher in these traits tend to be both psychologically and physiologically healthier and experience fewer negative emotions in stressful situations (26). However, some studies have contradicted these findings, suggesting that the relationship between personality traits and sleep quality may not be consistent across different contexts (22, 30). For example, Križan and Hisler (22) found no significant relationship between extraversion and objectively measured sleep quality, and Mead et al. (30) reported that extraversion may even negatively impact sleep quality.

Although research generally shows that agreeableness and openness are unrelated to sleep quality (21, 23, 30), Cellini et al. (10) found that when cognitive reappraisal, inhibition, emotions, and hyperarousal were included in the regression model, agreeableness was the only personality trait that predicted sleep quality. Spears et al. (9) also suggested that agreeableness may indirectly predict mortality risk by affecting daytime impairments and sleep. Furthermore, Leger et al. (26) found evidence suggesting that openness might be associated with better sleep quality, as individuals with high openness tend to experience fewer negative emotions and may, therefore, have better sleep quality.

### 1.2 Measuring personality and sleep from social media

Due to the limitations of traditional methods in large-scale data collection, real-time analysis, and cost efficiency, researchers have increasingly turned to social media in recent years to assess mental health indicators, leveraging its natural user expressions and traceable characteristics as complementary tools (31–35). These studies focused on three characteristics: behavioral features [e.g., number of posts, timing of posts, frequency of likes (31, 36), and comments on other posts (37)]; multimedia features [e.g., videos (38) and images (39)]; and text features. For text features, three semantic representation methods were mainly used to understand the content: discrete representations [e.g., term frequency-inverse document frequency (40)], closed vocabulary methods [e.g., Linguistic Inquiry and Word Count (41) and self-defined dictionaries (42)], and open vocabulary methods [e.g., Word2Vec (43) and BERT (44)]. Among these, open vocabulary methods using deep learning-based word embeddings provide the most effective representation of social media text (45).

Regarding personality, most research on personality assessment has focused on English text (46), with few studies examining the Chinese context using distributed representation methods for data training models. For example, Mahajan et al. (47) found that online-revealed personalities align with users’ true personalities. Cutler and Condon (48) conducted factor analysis on word embeddings of English text using BERT and compared the results with earlier lexical studies. They found that agreeableness, extraversion, and conscientiousness traits were well-replicated, but neuroticism and openness were not. Considerable research attention has focused on examining the relationship between sleep characteristics and their associated variables on social media platforms such as Twitter, Weibo, and Reddit (49). For example, Liu et al. (50) examined sleep-related user attributes like region and education levels, and Yao et al. (51) investigated how sleep quality as a symptom accompanies other mental health issues within a depression community, identifying co-occurrences with fear, negative expectations, and suicidal intentions. Relatively few studies have evaluated sleep problems using natural language processing techniques. For instance, Tian et al. (52) employed a support vector machine algorithm to detect sleep-related complaints in social media posts and identify key topics associated with insomnia.

### 1.3 Research objectives

Previous studies have established connections between personality traits and sleep characteristics, but inconsistent methodologies have produced mixed results (4). Two persistent issues complicate research in this area. First, data availability remains limited. Most existing datasets that include both sleep and personality measures are small and not publicly accessible, which restricts the generalizability of findings. Second, research approaches are often disconnected. Although social media platforms offer rich natural language data, most studies focus exclusively on either personality assessment or insomnia detection, with little attention to the interaction between the two (53).

This study aims to investigate the relationship between personality traits and both model-assessed sleep problems and self-reported sleep quality. Specifically, we focus on four main objectives:

1. Develop and validate deep learning models for assessing sleep problems.
2. Develop and validate deep learning models for personality assessments.
3. Examine how personality traits correlate with self-reported sleep quality in survey responses.
4. Examine the link between personality traits and model-assessed sleep problems in the large dataset.

## 2 Methods

### 2.1 Overview of the methodological process

**Figure 1:**
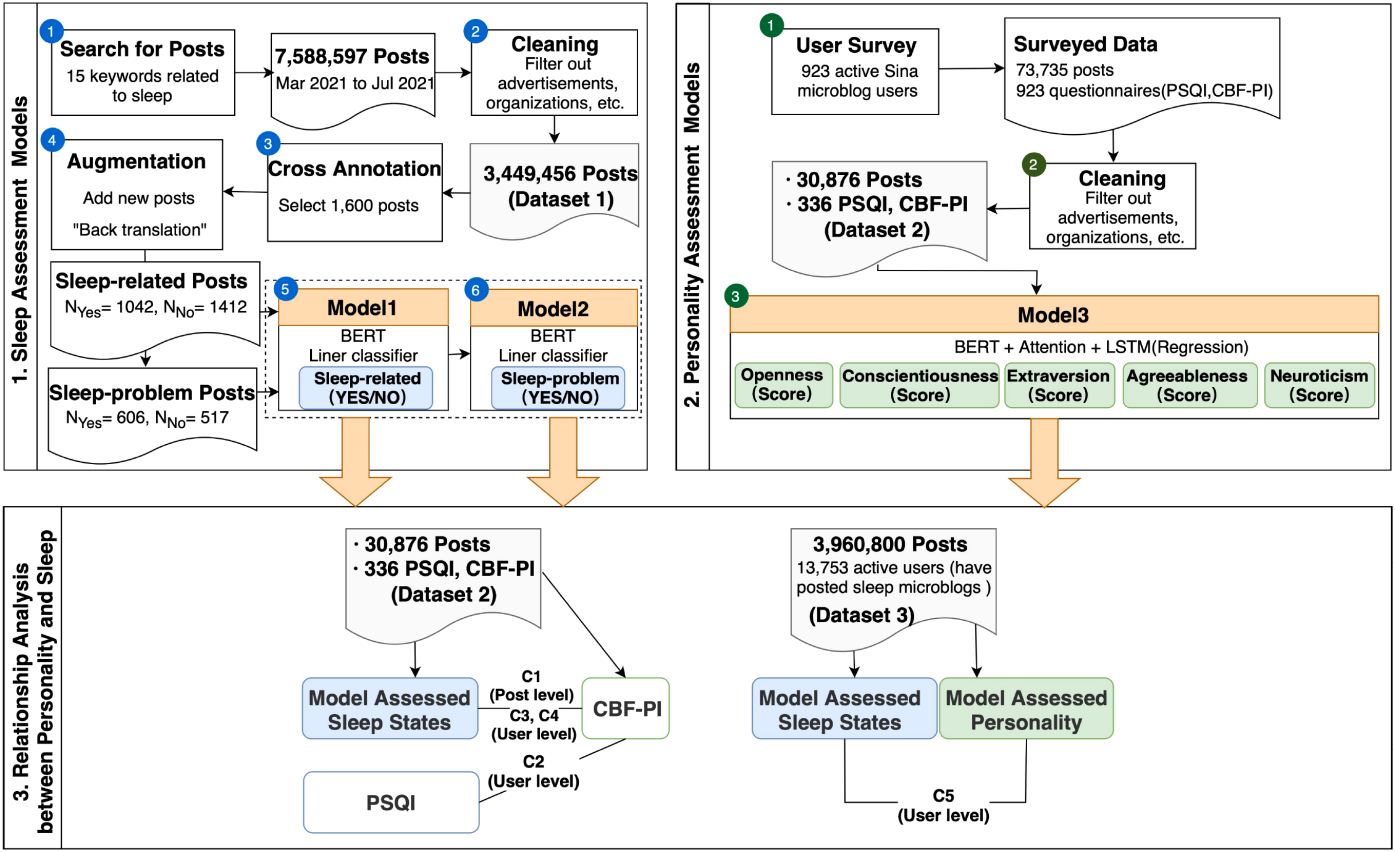
The framework of the study. CBF-PI, Chinese simplified big five personality inventory (54); PSQI, Pittsburgh sleep quality index (11).

As shown in **Figure 1**, we followed a systematic process for project design, data collection, and implementation. (1) First, we collected microblogs from Sina Weibo, China’s largest social media platform, and constructed two datasets. The first dataset was a large-scale collection, consisting of 4,860,000 posts from 15,251 users. The second dataset combined microblogs with survey responses from 923 Sina Weibo users (336 active users). For data collection and management, we used PyCharm 2021.3.1 (Community Edition, JetBrains) to process the microblog text. (2) We then built two sleep assessment models: Model 1 was designed to determine whether a post was sleep-related, and Model 2 assessed whether a post indicated a sleep problem. For personality assessment, we applied BERT-based word embeddings in combination with long short-term memory (LSTM) (55) regression models incorporating an attention mechanism (Model 3). (3) To analyze the relationship between personality traits (FFM) and sleep characteristics, we used both self-reported data and model-generated assessments. As part of an exploratory analysis, we applied the three semantic models, which independently assess personality traits and sleep problems, to the large-scale microblog dataset to further investigate their relationship in this context.

### 2.2 Data collection

#### 2.2.1 Microblog dataset

The construction of the microblog dataset followed four steps: collection, cleaning, encoding, and enhancement. We collected microblogs from March 2021 to July 2021 using a set of sleep-related keywords describing sleep-related issues (e.g., “insomnia”, “stay up”, “extensive dream”, “nightmare”, “startle awake”, “early morning”, “unable to sleep” and “sleepy”). Thus, we obtained a large microblog dataset (N = 7,588,597) potentially reflecting sleep problems. From this dataset, we randomly selected a smaller subset for human labeling. The dataset had an average of 219.46 posts per user.

##### 2.2.1.1 Text cleaning and encoding

We counted the frequency of stop words (e.g., advertisement and marketing account) and filtered noisy posts via manual screening. This resulted in 3,449,456 posts for subsequent analysis. We randomly selected 1,600 posts, and each post was labeled in two steps by two undergraduate psychology students. For the label of “whether the post is related to sleep”, a total of 1,497 posts were consistently labeled (r = 0.84, *p <*.001), of which 1,042 posts were sleep-related. For the label of “whether the post reflects a sleep problem”, a total of 774 posts were labeled consistently (r = 0.41, *p <*.001), of which 606 posts were related to a “sleep problem”. In the subsequent model building, we only used consistently labeled data.

##### 2.2.1.2 Data enhancement

To balance the sample size for the training set, we applied different strategies to the training set of Model 1 (“whether the post is related to sleep”) and Model 2 (“whether the post reflects a sleep problem”). For Model 1, we added 957 posts that did not include sleep-related keywords and were not related to sleep. For Model 2, we used the back-translation method (using the googletrans library in Python): the original post was translated into English, then translated into Spanish, and finally translated back into Chinese. We obtained 517 posts that did not express sleep problems, and 1,123 posts were selected for training Model 2.

#### 2.2.2 User survey

##### 2.2.2.1 Participants

The surveys were created using Qualtrics and distributed in two ways: 1) alongside the “# Questionnaire Mutual Filling” super topics on Sina Weibo, and 2) via the PsyExperimentor participant recruitment platform ^1^. A total of 923 questionnaires were collected, of which 336 were valid and from active users. The average age of participants was 23.66 years (standard deviation (*SD*) = 4.60), and 257 were women. Participants were excluded if they failed the lie detection questions, were inactive microblog users (i.e., had fewer than five original posts), or had nonexistent or marketing-focused user identifications (ID). Through the user IDs, we crawled the original posts of all participants from January 2020 to January 2023 (N = 73,735). On average, each participant made 219.45 original posts. The highest and lowest number of posts by a participant was 3,945 and 5, respectively.

##### 2.2.2.2 Questionnaires

The questionnaire comprised two parts: (1) a basic information questionnaire, which included age, gender, household registration, occupation, frequency of usage, nickname, and user ID; and (2) the Chinese Simplified Big Five Personality Inventory (CBF-PI) (54). The CBF-PI comprises a total of 15 items, with three items for each of the five personality traits. Responses are made on a six-point scale, with responses ranging from 1 (completely disagree) to 6 (completely agree). Two items are scored in reverse. Cronbach’s alpha coefficients for openness, conscientiousness, extraversion, agreeableness, and neuroticism were 0.82, 0.72, 0.82, 0.83, and 0.77, respectively. The descriptive statistics of the valid users (N = 336) for each dimension of the FFM are presented in **Supplementary Table 1**. The scores for each dimension were normally distributed. The PSQI (11) comprises 19 items across seven dimensions, and each item is scored on a four-point scale. The total score represents sleep sleep quality, with higher scores indicating poorer sleep quality. Excluding five fill-in-the-blank questions, Cronbach’s alpha coefficient for the PSQI was 0.84.

### 2.3 Sleep assessment models

We developed two sleep assessment models. The first model assessed each post on “whether it is related to sleep” (Model 1). For posts that are assessed as being “related to sleep”, the second model evaluated “whether there are sleep problems” (Model 2). Models 1 and 2 shared a similar structure, and both utilize a BERT fine-tuning approach with a downstream fully connected neural network to build a classifier. To ensure computational efficiency, the text was first segmented into sentences. Due to BERT’s input limitation of 512 characteristics, sentences exceeding this length were truncated. After segmentation, each sentence was first passed through a pre-trained BERT Chinese model to convert each word into word embeddings, which were subsequently input into a Transformer Encoder (*Trm*). The *Trm* established semantic relationships between words according to the context. Then the output from the *Trm* was then fed into four types of classification models: a linear fully connected layer (BERT + Linear), a convolutional neural network (BERT + CNN), a recurrent neural network (BERT + LSTM), and a recurrent neural network with an attention mechanism (BERT + LSTM + Attention). These models were chosen to assess different types of contextual and sequential relationships within the text, allowing us to test various architectures for better performance.

To further evaluate the performance and robustness of our models, we also converted each sentence into word embeddings using a pre-trained ERNIE model [Enhanced Representation through Knowledge Integration; (56)], which was then input into a linear classifier (ERNIE + Linear). This additional model served as a comparative baseline, allowing us to assess the impact of using a different pre-trained language model for the task. The results from these models were compared to determine the most effective architecture for assessing sleep-related content and sleep problems in microblog posts, providing insights into which approach best captured the nuances of sleep-related language.

We split the labeled dataset for Model 1 (N = 2,454) and the labeled dataset for Model 2 (N = 1,123) into training, validation, and testing sets using a ratio of 8:1:1. Specifically, 80% of the data was used for training the model, 10% for model validation, and the remaining 10% for testing. This approach ensured that the models were trained on a sufficient amount of data while retaining a separate dataset for testing performance and tuning. To evaluate the performance of the models, we used Precision (P), Recall (R), and F1 score as key metrics. These metrics were chosen because they offer a balanced view of model performance. In our evaluation, the F1 score was prioritized because it combines both Precision and Recall into a single metric, offering a more comprehensive understanding of the model’s performance.

### 2.4 Personality assessment models

**Figure 2:**
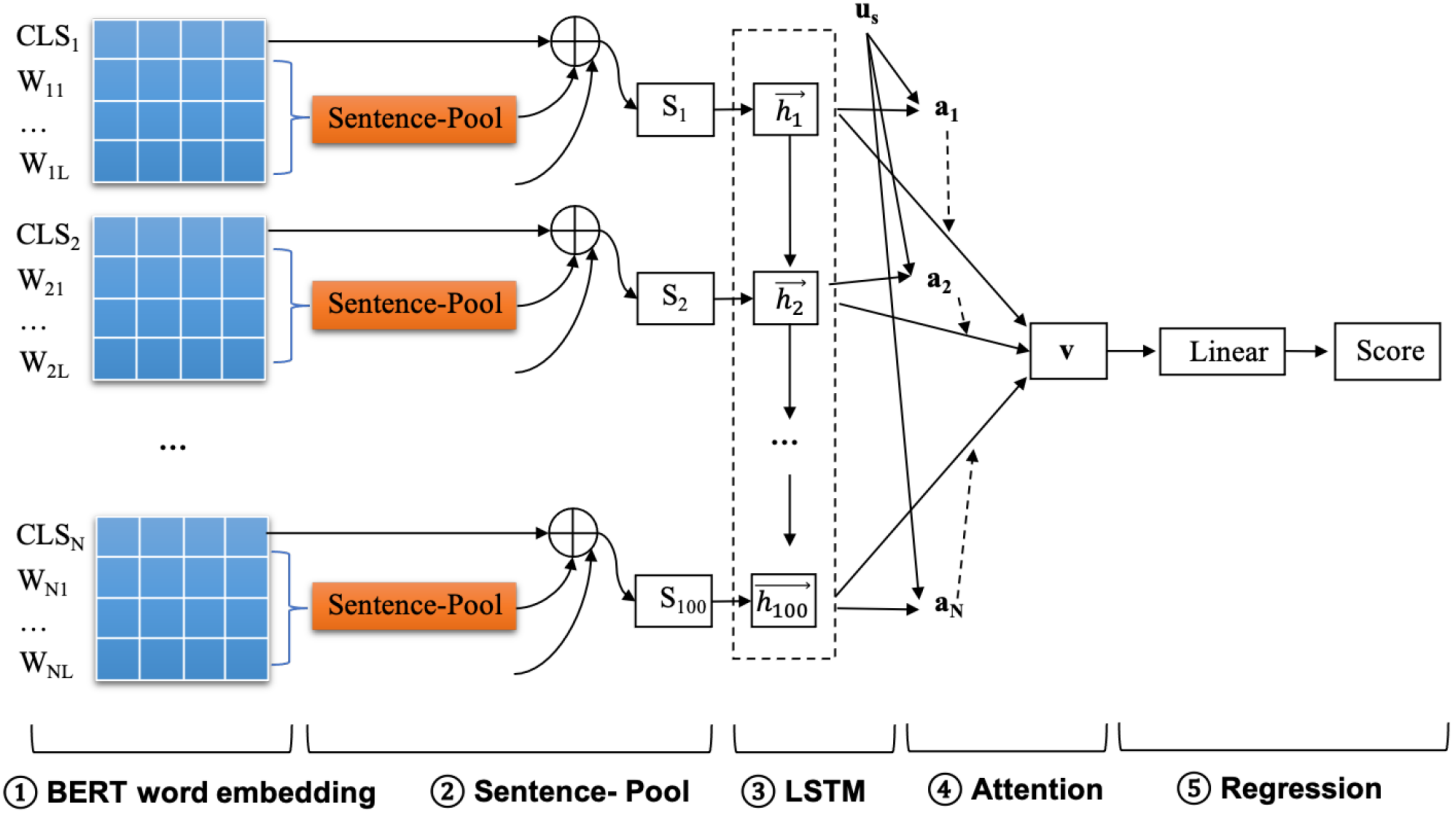
Structure of the personality assessment models (Model 3). Each model contains five layers: bidirectional encoder representations from transformers (BERT) word-embedding layer, sentence fusion layer, LSTM layer, attention layer, and regression layer. We trained five separate regression models for the Big Five personality traits. We used users’ self-reported scores as labeled inputs for training.

The personality assessment model based on microblog text comprised five components: the BERT embedding layer, sentence fusion layer, LSTM layer, attention layer, and regression layer (**Figure 2**). This model evaluated personality as a trait variable, assuming it remains stable over time. Therefore, we aggregated multiple posts from each user to form a sequence of text data, which was crucial for capturing the consistency of the user’s personality expression over time. Initially, word embeddings for each post were generated using the Chinese BERT pre-trained model. Since BERT had an input limitation of 512 tokens, posts longer than this threshold were truncated, and a “split then merge” approach was applied to ensure that the word embeddings accurately represented longer posts. In the sentence fusion layer, the word embeddings from the most recent 100 posts were selected for subsequent training. For users with fewer than 100 posts, zero vectors were used for padding. This resulted in each user’s word embeddings being represented as a 100 × 768 matrix. The sequence of word embedding matrices for each user was then input into the LSTM layer. LSTM, a specialized type of recurrent neural network, was particularly suited for handling sequence data. Here, each user’s posts were treated as a sequence, allowing the LSTM to capture the temporal dynamics and semantic relationships across the multiple posts of each user. By processing these sequences, the model could learn how a user’s personality traits were reflected and expressed over time in the context of their social media posts.

To enhance the model’s focus on relevant personality-related features, we incorporated an attention mechanism. This mechanism allocated attention to different parts of the sentence vectors, enabling the model to better capture key personality-related content from each post, thereby improving classification accuracy. Finally, a linear regression model was applied to the output of the attention layer to generate the personality scores.

We trained five separate regression models for the Big Five personality traits, using normalized selfreported CBF-PI scores as labeled inputs. Normalizing personality scores improved training efficiency, reduced the impact of data sparsity and outliers, and led to a more stable model. In addition to the primary model, we compared the BERT-based model’s performance with simpler models such as a linear fully connected layer (BERT + Linear) and a recurrent neural network (BERT + LSTM). The effectiveness of these models was evaluated by comparing the predicted personality scores with the labeled values using Pearson’s correlation coefficient and RMSE. This comparison allowed us to assess the relative performance of different models in capturing personality traits from user-generated microblog content.

## 3 Results

### 3.1 Development and validation of deep learning models for sleep problem assessment

The results of the sleep assessment models (i.e., Models 1 and 2) and comparisons with alternate models are shown in **Table 1**. When determining whether a post is related to sleep (Model 1), the best performance was achieved when using only the BERT fine-tuning approach (accuracy = 95.09%, precision = 97.27%, F1 score = 0.95). When determining whether a sleep-related post indicated a sleep problem (Model 2), the best performance was achieved when using only the BERT fine-tuning approach, which had an accuracy of 91.30%, a precision of 91.65%, and an F1 score of 0.91.

**Table 1:** Results of sleep assessment models: Model 1 assessed whether a post is related to sleep, and Model 2 evaluated whether there is a sleep problem in the post.

Model	Model 1			Model 2		
	Precision	Recall	F1	Precision	Recall	F1
BERT + Linear	95.09%	**97.27%**	**0.95**	**91.30%**	**91.65%**	**0.91**
ERNIE + Linear	**97.25%**	92.17%	0.95	90.20%	88.46%	0.89
BERT + CNN	88.60%	87.83%	0.88	86.27%	84.62%	0.85
BERT + LSTM	92.52%	90.00%	0.91	89.36%	80.77%	0.85
BERT + LSTM + Attention	89.74%	91.30%	0.91	62.67%	90.38%	0.74

Bold values indicate the best model performance.

It is noteworthy that the assessment models for sleep-related and sleep problem posts, which used only BERT or ERNIE fine-tuning followed by a fully connected neural network output, performed better than those that incorporated additional deep learning algorithms. This indicates that, for our tasks, fine-tuning pre-trained models is sufficient to achieve good classification results. In fact, the addition of more complex models tended to degrade performance. This aligns with the findings of Mohammadi and Chapon (57), who demonstrated that when fine-tuning BERT pre-trained models, fully connected neural networks perform better than more complex classifiers for text classification tasks. Simpler classifiers can maximize the use of BERT’s text representation capabilities, resulting in better performance, whereas complex classifiers are more prone to issues like overfitting, which can degrade performance.

We applied the sleep assessment models (Model 1 and Model 2) to a total of 73,735 posts from 336 valid participants. Among these, 2,709 posts were identified as related to sleep, and 1,578 of them were further identified as expressing sleep problems. For each participant, we calculated: (1) the total number of posts (TN, i.e., posting frequency); (2) the number of sleep-related posts (NSR); (3) the number of posts indicating sleep problems (NSP); (4) the proportion of sleep-related posts (PSR); and (5) the proportion of posts with sleep problems (PSP). Among the 336 users, 192 posted sleep-related content (with a maximum of 168 such posts and a highest proportion of 53.30%), and 172 users posted content expressing sleep problems (with a maximum of 81 such posts and a highest proportion of 53.33%).

**Figure 3:**
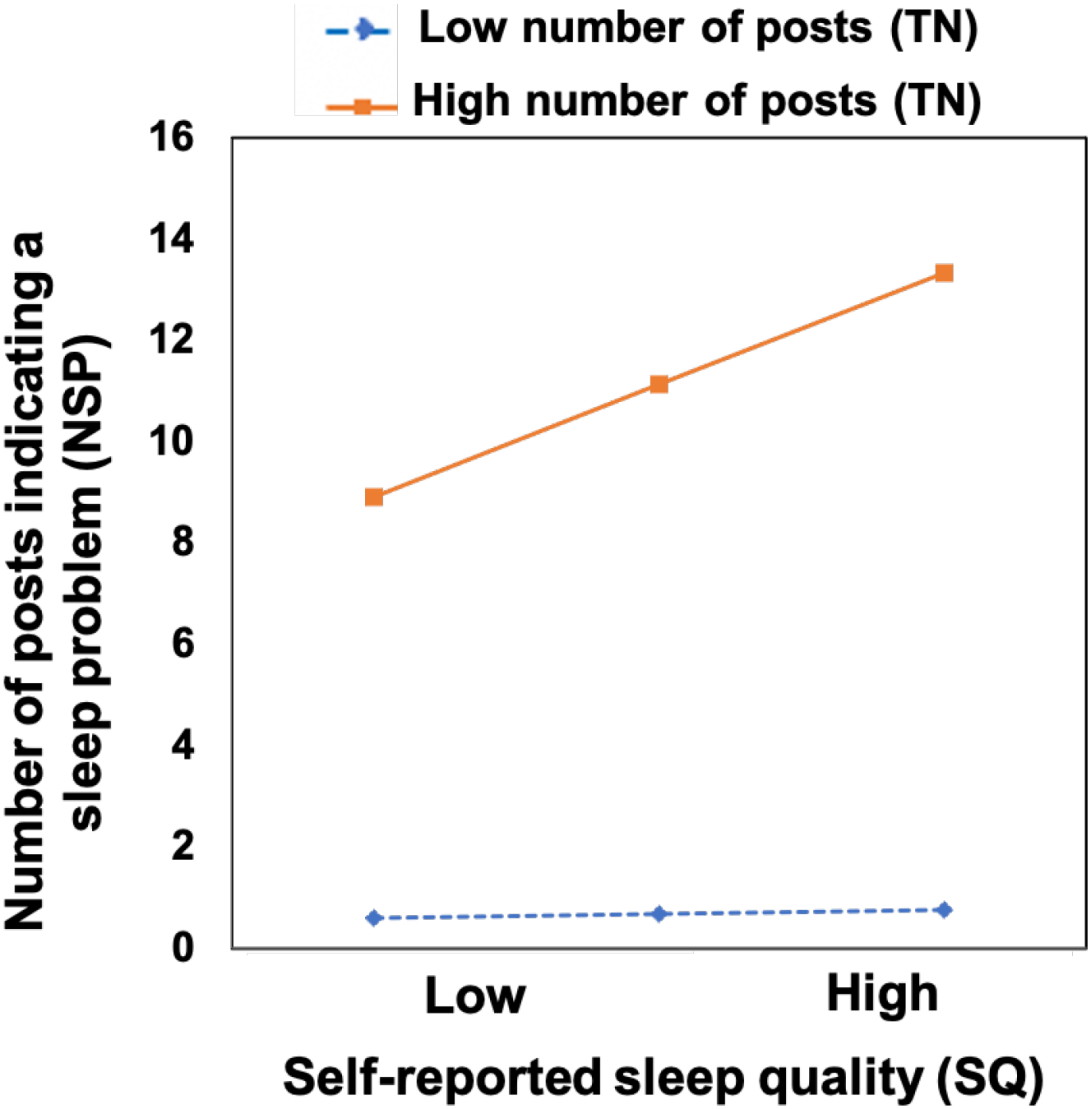
The moderating effect of the total number of posts (TN) on self-reported sleep quality (SQ, assessed using the pittsburgh sleep quality index (PSQI)) and the number of posts indicating a sleep problem (NSP, assessed using Model 2).

We initially used self-reported sleep quality (i.e., PSQI scores) to evaluate the validity of sleep characteristics assessed by Model 1 and Model 2. However, PSQI scores were not significantly correlated with any of the four model-assessed sleep characteristics. To further explore potential influencing factors, we examined the correlation between the number of posts indicating sleep problems (NSP) and the total number of posts (TN). A strong positive correlation was observed (Pearson’s r = 0.64, *p <*.001), indicating possible collinearity. To address this, we conducted a moderation analysis with PSQI score as the independent variable, the number of posts indicating sleep problems (NSP) as the dependent variable, and the total number of posts (TN) as a moderating variable. Age and gender were included as covariates. The results showed that poorer self-reported sleep quality (i.e., higher PSQI scores) significantly predicted a greater number of sleep problem posts (NSP) (*β* = 0.68, *p* = .015). In addition, the interaction between sleep quality and the total number of posts (TN) was significant (*β* = 0.12, *p* = .006), suggesting a moderating effect of user activity. Simple slope analysis (**Figure 3**) indicated that when the posting frequency was low, the PSQI score did not significantly predict the number of sleep problem posts (NSP) (*β* = 0.06, *p* = .817). However, at a high level of posting frequency, higher PSQI score significantly predicted more posts expressing sleep problems (*β* = 1.49, *p <*.001). A similar pattern was observed for the number of sleep-related posts (NSR). From these findings, we conclude that:

Conclusion 1: Self-reported sleep quality significantly predicts model-assessed sleep problems, and this relationship is moderated by users’ total posting frequency.

### 3.2 Development and validation of deep learning models for personality assessments

The predictive performance of the personality (Big Five) models (Model 3) is shown in **Table 2**. When the input consisted of sentence vectors extracted using BERT features, the LSTM regression model based on the attention mechanism performed the best (average RMSE = 0.186), with predicted values for the test set significantly positively correlated with the questionnaire scores. Using the linear regression model or the LSTM model separately resulted in poor performance, with low, non-significant correlation coefficients between the predicted and questionnaire scores and higher RMSE. It is noteworthy that the performance of the personality assessment model (Model 3) varied across the five traits. Openness (*r* = 0.50, *p* = .003) and conscientiousness (*r* = 0.51, *p* = .003) performed well, with correlations around 0.5, whereas extraversion (*r* = 0.27, *p* = .021), agreeableness (*r* = 0.33, *p* = .013), and neuroticism (*r* = 0.24, *p* = .047) showed poorer performance. The self-reported questionnaires indicated generally high scores, with uneven training data for agreeableness. Results for agreeableness and extraversion may also have been affected by social desirability factors, such as acceptance, pro-social behavior, and sociability (58). Furthermore, agreeableness, which is a highly evaluative trait, may lead to inaccuracies in self-assessment and, in turn, inconsistent results (59).

**Table 2:** Results of personality assessment models: the Chinese Simplified Big Five Personality Inventory (CBF-PI) as a measure of FFM.

Method	Result	O	C	E	A	N
CBF-PI Score	*M*	10.82	13.34	10.12	13.80	10.34
	*SD*	3.23	2.54	3.40	2.64	3.28
BERT + Linear	*r*	-0.04	0.09	0.04	0.04	-0.30
	RMSE	0.34	0.28	0.38	0.25	0.42
BERT + LSTM	*r*	0.14	0.09	0.10	0.20	0.14
	RMSE	0.21	0.19	0.22	0.19	0.24
BERT + LSTM + Attention	*r*	0.50**	0.51**	0.28**	0.33*	0.24*
	RMSE	**0.17**	**0.16**	**0.21**	**0.17**	**0.22**

O, Openness; C, Conscientiousness; E, Extraversion; A, Agreeableness; N, Neuroticism; M, Mean; SD, Standard Deviation; *r*, Pearson’s correlation coefficient; RMSE, Root Mean Squared Error. *p <.05, **p <.01.

Bold values indicate the best model performance.

### 3.3 Relationship between personality traits and self-reported sleep quality

We examined the correlations between self-reported sleep quality (i.e., PSQI score), the number of sleep-related posts (NSR), the number of posts indicating a sleep problem (NSP), and the proportion of posts with sleep problems (PSP) and personality traits (measured by CBF-PI scores) (**Supplementary Table S1** in **Supplemental Materials**). The results showed significant associations between all five personality traits (openness, conscientiousness, extraversion, agreeableness, and neuroticism) and PSQI scores. Then we used these four model-assessed sleep characteristics as dependent variables and other variables (i.e., personality traits, gender and age) that showed significant correlations with these characteristics as independent variables in the regression model. Results in **Table 3** show that agreeableness significantly predicted better self-reported sleep quality (*β* = 0.14, *p <*.01), while neuroticism significantly predicted poorer self-reported sleep quality (*β* = 0.4, *p <*.001). Therefore, we concluded that:

**Table 3:** Regression analysis of personality traits (CBF-PI scores) with PSQI and model-assessed sleep characteristics in 336 users.

Regression models		Model fit					
Dependent	Independent	*R*	*R* ^2^	*F*	*B* (95% CI)	*β*	*t*
PSQI	O	0.53	0.27	26.35**	0.03 [–0.06,0.13]	0.04	0.49
	C				–0.11 [–0.24,0.01]	–0.10	–1.85
	E				–0.07 [–0.17,0.02]	–0.08	–1.50
	A				–0.15 [–0.27,0.46]	–0.14	–2.70*
	N				0.36 [0.27,0.46]	0.40	7.61***
NSR	Age	0.20	0.03	3.50*	0.37 [–0.04,0.78]	0.10	1.76
	O				–0.33 [–1.72,–0.08]	–0.06	–0.96
	C				–0.90 [–1.72,–0.08]	–0.13	–2.15*
	E				–0.24 [–0.87,0.39]	–0.05	–0.74
PSR	Gender	0.26	0.05	5.75**	3.14 [0.53,5.74]	0.13	2.37*
	Age				0.26 [0.02,0.49]	0.12	2.16*
	O				–0.28 [–0.64,0.07]	–0.09	–1.57
	C				–0.40 [–0.82,0.06]	–0.10	–1.71

O, Openness; C, Conscientiousness; E, Extraversion; A, Agreeableness, N, Neuroticism; PSQI, Pittsburgh Sleep Quality Index; NSR, Number of Sleep-Related Posts; PSR, Proportion of Sleep-Related Posts; PSP, Proportion of Posts with Sleep Problems; R, Pearson’s correlation coefficient; R², Coefficient of Determination; F, F-statistic (used to assess model fit); 95% CI, 95% Confidence Interval; B, Unstandardized regression coefficient; *β*, Standardized regression coefficient; t, t-statistic (used for hypothesis testing). *p<.05, **p<.01.

Bold values indicate statistically significant results.

Conclusion 2: Openness, conscientiousness, extraversion, and agreeableness were significantly associated with better self-reported sleep quality, while neuroticism was linked to poorer self-reported sleep quality in the correlation analysis. Agreeableness and neuroticism remained significant predictors in the multivariate regression.

### 3.4 Relationship between personality traits and model-assessed sleep problems

We applied the sleep assessment models (i.e., Models 1 and 2) to the dataset comprising 73,735 posts from 336 surveyed users. We then explored the relationships between sleep characteristics and personality traits at both the post and user levels to provide a comprehensive view.

**Figure 4:**
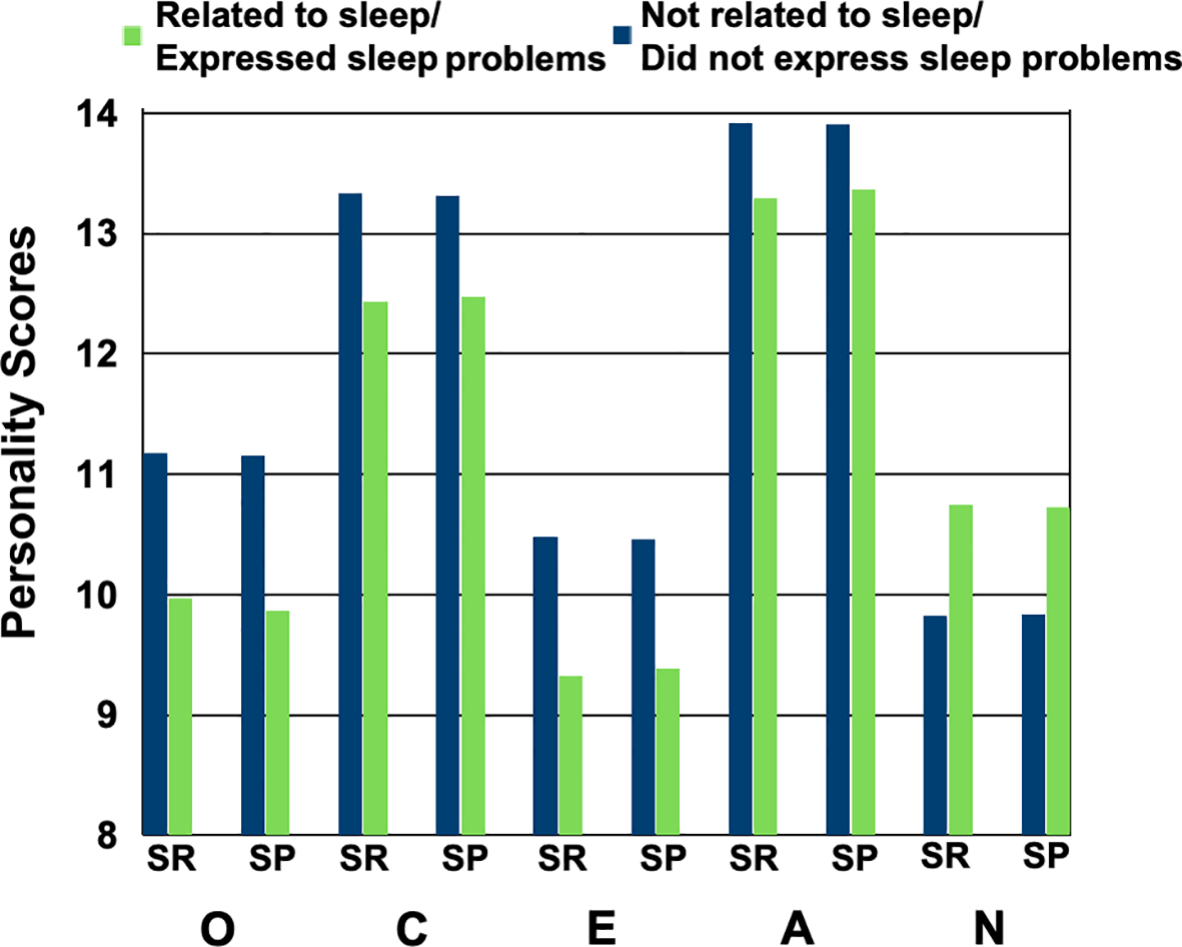
Relationship between sleep and the big five personality traits (openness, conscientiousness, extraversion, agreeableness, and neuroticism) at the post level (tested on the user survey dataset). “SR” is an abbreviation for “Sleep-Related”, indicating whether the post is related to sleep; “SP” is an abbreviation for “Sleep Problems”, indicating whether the post explicitly expresses sleep problems. The green bars represent posts classified as “Related to sleep” or “Expressed sleep problems”, and the blue bars represent posts classified as “Not related to sleep” or “Did not express sleep problems”.

At the post level, “whether a post is related to sleep (SR)” and “whether a post indicates a sleep problem (SP)” were used as independent variables, and self-reported personality traits (CBF-PI scores) were used as dependent variables in independent samples t-tests. Due to the small proportion of sleep-related posts for all personality traits except agreeableness, we applied corrections for unequal variances using IBM SPSS Statistics for Mac (Version 26.0.0.2). The results are shown in **Figure 4**. We found two key results: (1) Posts related to sleep (SR) had significantly lower scores in openness (*t* = 20.88, *p <*.001), conscientiousness (*t* = 19.23, *p <*.001), extraversion (*t* = 17.16, *p <*.001), and agreeableness (*t* = 11.67, *p <*.001), and had significantly higher scores in neuroticism (t = 17.07, p *<*.001); (2) Compared to posts without sleep problems, posts expressing sleep problems (SP) had significantly lower scores in openness (*t* = 17.52, *p <*.001), conscientiousness (*t* = 13.99, *p <*.001), extraversion (*t* = 12.47, *p <*.001), and agreeableness (*t* = 7.70, *p <*.001), and significantly higher scores in neuroticism (*t* = −12.93, *p <*.001). Therefore, we drew the following conclusion:

Conclusion 3: Openness, conscientiousness, extraversion and agreeableness measured by CBF-PI scores are significantly associated with fewer model-assessed sleep problems, whereas neuroticism is significantly was associated with more model-assessed sleep problems at the post level.

At the user level, we first examined the correlations between personality traits (measured by CBF-PI scores) and the four model-assessed sleep characteristics (the number of sleep-related posts (NSR), the proportion of sleep-related posts (PSR), the number of posts indicating a sleep problem (NSP) and the proportion of posts with sleep problems (PSP)) (**Supplementary Table S1** in Supplemental Materials). The number of sleep-related posts (NSR) was significantly negatively correlated with openness (*r* = −0.12, *p* = .031), conscientiousness (*r* = −0.16, *p* = .006), and extraversion (*r* = −0.11, *p* = .046). The number of posts indicating a sleep problem (NSP) was significantly negatively correlated with openness (*r* = −0.14, *p* = .013) and conscientiousness (*r* = −0.16, *p* = .003). The proportion of posts with sleep problems (PSP) was significantly negatively correlated with openness (*r* = −0.14, *p* = .013; **Supplementary Table S1**). Further regression analysis revealed that conscientiousness was a significant negative predictor of the number of sleep-related posts (NSR) (*β* = −0.13, *p* = .032).

Further, we explored the relationship between model-assessed sleep characteristics and model-assessed personality traits from a big data-driven perspective. We randomly selected 15,251 users who had posted sleep-related content and crawled all of their original posts (N = 4,864,600) made between January 2020 to January 2023 using the user IDs. We then selected users who had posted more than five original posts (N = 13,753; 11,035 women, 2,627 men, and 91 with undisclosed gender), resulting in a total of 3,960,000 posts. We applied the sleep assessment models (i.e., Models 1 and 2) and the personality assessment model (Model 3). Results showed that 12,623 users had written sleep-related posts, which totaling 447,600 posts (11.3% of all posts), and 12,274 users had written about sleep problems, which totaled 332,600 posts (8.4% of all posts). Based on the analysis results from the surveyed dataset, we used two model-assessed sleep characteristics: the number of sleep-related posts (NSR) and the number of posts indicating a sleep problem (NSP), and conducted a correlation analysis between these sleep characteristics and personality traits. As shown in **Table 4**, the number of sleep-related posts (NSR) was significantly correlated with lower scores in conscientiousness (*r* =−0.07, *p <*.001), extraversion (*r* =−0.16, *p <*.001), and agreeableness (*r* =−0.14, p *<*.001), and significantly correlated with higher scores in neuroticism (*r* =0.23, p *<*.001). For the number of posts indicating a sleep problem (NSP), the results were similar to those for the number of sleep-related posts (NSR), with the addition of a significant positive correlation with openness (*r* = 0.02, *p* = .027).

**Table 4:** Correlations between model-assessed sleep characteristics and personality traits.

Sleep Characteristics	Model-assessed personality traits				
	O (95% CI)	C (95% CI)	E (95% CI)	A (95% CI)	N (95% CI)
NSR	0.01 [−0.01,−0.02]	−0.07^∗∗∗^ [−0.09,−.05]	−0.16^∗∗∗^ [−0.18,−0.14]	−0.14^∗∗∗^ [−0.15,−0.12]	0.23^∗∗∗^ [0.21,0.24]
NSP	0.02^∗^ [0.01,0.04]	−0.06^∗∗∗^ [−0.07,−0.04]	−0.16^∗∗∗^ [−0.18,−0.15]	−0.15^∗∗∗^ [−0.17,−0.14]	−0.20^∗∗∗^ [−0.18,−0.22]

Users = 13,753.

O, Openness; C, Conscientiousness; E, Extraversion; A, Agreeableness; N, Neuroticism; NSR, The number of sleep-related posts; NSP, The number of posts indicating a sleep problem; 95% CI, 95% Confidence Interval; *p <.05, ***p <.001.

We analyzed the model-assessed personality scores in the large dataset and found two key issues: (1) The standard deviation (SD) of model-assessed personality trait scores was lower than that of self-reported scores in the surveyed dataset (N = 336; **Supplementary Tables S2**, **S3**). Since the model’s training goal was to minimize the loss function, predictions tended to cluster around the mean value. (2) There was collinearity among the model-assessed personality traits, likely due to the use of the same dataset for training the models of the five dimensions. Despite these issues, the model-assessed personality traits was significantly correlated with the self-reported personality traits (**Table 2**), indicating that the relative magnitude of each user’s personality scores reflected their position within the entire group.

**Figure 5:**
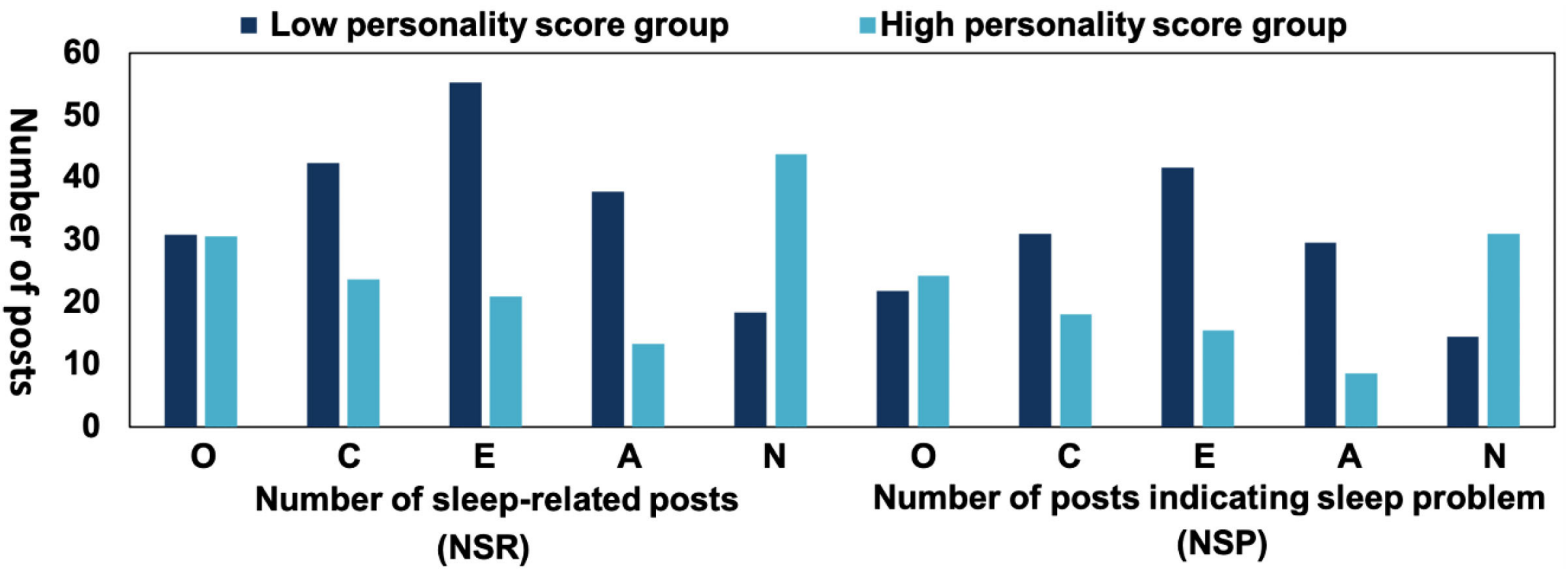
Differences in the number of posts [the number of sleep-related posts (NSR) and the number of posts indicating a sleep problem (NSP)] between users with high and low scores for the various personality traits (N = 13,753). O, Openness; C, Conscientiousness; E, Extraversion; A, Agreeableness; N, Neuroticism.

To address these two issues, we ranked and grouped the model-assessed personality traits (N = 13,753) and selected the top and bottom 27% of the data to form low-score and high-score groups, respectively, for each dimension. The 27% selection was chosen to ensure a clear distinction between the low-score and high-score groups, capturing significant extremes while minimizing overlap and potential collinearity from the central range. **Figure 5** shows that: (1) users in the high-score group for conscientiousness (*t* = 14.96, *p <*.001), extraversion (*t* = 23.36, p *<*.001), and agreeableness (*t* = 19.65, *p <*.001) had significantly fewer sleep-related posts than those in the low-score group for these traits, while users in the high neuroticism group had significantly more sleep-related posts than those in the low neuroticism group (*t* = −21.02, *p <*.001); (2) users in the high-score group for openness (*t* =−2.18, *p* = .029) and neuroticism (*t* =−17.54, *p <*.001) had significantly more posts indicating a sleep problem than those in the low-score group for these traits, while users in the high-score group for conscientiousness (*t* = 11.42, *p <*.001), extraversion (*t* = 22.68, *p <*.001), and agreeableness (*t* = 21.58, *p <*.001) had significantly fewer posts indicating a sleep problem than those in the low-score group for these traits. Thus, we concluded that:

Conclusion 4: From a big data-driven perspective, higher model-assessed sleep characteristics were significantly associated with lower scores in model-assessed conscientiousness, extraversion, and agreeableness, and with higher scores in neuroticism. These findings largely align with the results from self-reported data.

## 4 General discussion

In this paper, we present a novel framework that integrates user surveys and big data-driven computational methods to assess sleep characteristics and personality traits from microblogs. Specifically, we constructed two classifiers based on BERT word embeddings to capture the deep semantic content of microblogs, enabling us to determine whether posts were related to sleep and whether they expressed sleep problems (Models 1 and 2, respectively). For the more complex task of personality assessment, we built a semantic feature space by fine-tuning BERT word embeddings and introduced an LSTM neural network with an attention mechanism to predict scores for the five personality traits manifested in the microblogs (Model 3). We collected an empirical dataset based on user surveys (total users = 923, active users = 336) and applied expert cross-annotation and self-reported questionnaires as the gold standard for training the three models. Our approach achieved high performance in assessing sleep characteristics and personality traits in both the training and testing datasets. We then applied these models to a separate large-scale microblog dataset (13,753 users and 4,864,600 posts).

Notably, our approach also scrutinized the reliability of big data methods in assessing psychological variables and their interrelationships. We analyzed these relationships from bothe survey-based and big data-driven perspectives and identified the commonalities with, and differences between, the findings of previous research across the five personality dimensions (**Table 5**). Previous research has consistently found a negative impact of neuroticism and a positive impact of conscientiousness on sleep quality (10, 21, 24). Furthermore, extraversion is also often associated with better sleep quality (9, 10, 21, 23). In the surveyed dataset, we found that:

1. Self-reported sleep quality significantly predicts model-assessed sleep problems, and this relationship is moderated by users’ total posting frequency.
2. Openness, conscientiousness, extraversion, and agreeableness measured by CBF-PI scores are significantly associated with better self-reported sleep quality, whereas neuroticism significantly predicts poorer self-reported sleep quality.
3. Openness, conscientiousness, extraversion and agreeableness measured by CBF-PI scores are significantly associated with fewer model-assessed sleep problems, whereas neuroticism is significantly was associated with more model-assessed sleep problems at the post level.
4. Self-reported sleep quality (as measured by the PSQI) predicted the number of sleep-related posts and the number of posts indicating a sleep problem, moderated by the total number of posts.
  When the models were applied to large-scale dataset, we found that:
5. Higher model-assessed sleep characteristics were significantly associated with lower scores in modelassessed conscientiousness, extraversion, and agreeableness, and with higher scores in neuroticism.

**Table 5:** Comparison of the personality–sleep relationship between our study and previous studies.

Author	Method	Participants	N	E	C	O	A
This study	Model1,2 vs. CBF-PI Self-reported sleep vs. CBF-PI	Posts (N = 73,735) Users (N = 336)	Negative Negative (predicted)	Positive Positive	Positive Positive	Positive Positive	Positive Positive (predicted)
	Model1,2 vs. CBF-PI	Users (N = 336)	–	Positive	Positive (predicted)	Positive (predicted)	–
Hintsanen et al. (60)	Model1,2 vs. Model3	Users (N = 13,753)	Negative	Positive	Positive	–	Positive
	Survey	Cross-culture (N=2,727)	Negative	Positive	Positive	–	Positive
Cellini et al. (10)	Survey	Subjects (N = 498)	Negative	Positive	Positive	–	Positive
Stephan et al. (21)	Survey (Longitudinal)	Four groups (N¿22,000)	Negative	Positive	Positive	–	–
Križan and Hisler (22)	Survey + ActiGraph	Adults (N = 382)	Negative	x	Positive	x	x
Sutin et al. (23)	Diary + ActiGraph	Adults (N = 620)	Negative	Positive	Positive	x	–
Spears et al. (9)	Survey (Longitudinal)	Users (N=3,759)	Negative	Positive	Positive	–	Negative
Mead et al. (30)	ActiGraph	College subjects (N = 358)	–	Negative	Positive	–	–

Model 1 assessed whether a post was related to sleep, and Model 2 assessed whether a post indicated a sleep problem. Model 3 assessed FFM personality traits. ““ indicates “not related”, and “x” indicates “inconsistent.”.

O, Openness; C, Conscientiousness; E, Extraversion; A, Agreeableness; N, Neuroticism; CBF-PI, The Chinese Simplified Big Five Personality Inventory as a measure of FFM; Self-reported sleep, PSQI score.

In regard to the automated assessment of psychological indicators from large-scale semantics, two issues are worth discussing. The first issue is the difficulty of manual text annotation and its impact on model performance. In our study, annotations showed high consistency for labeling “sleep-related” posts but low consistency for labeling “sleep problems” due to the complexity of the task. Identifying sleep-related content is straightforward using keywords like “sleep” and “stay up late”, whereas assessing sleep problems requires consideration of context and emotions. We initially attempted to annotate the “causes” and “manifestations”. However, due to data sparsity (i.e., lack of explicit causes) and complexity (i.e., vague expressions or mixed causes), most annotators labeled the text as “unable to determine”, leading to a small and low-quality dataset. Consequently, these annotations were not used for further study. For complex psychological indicators, future work could improve training data accuracy and richness by increasing the amount of annotated data, standardizing the annotation process, and recruiting experienced annotators.

Second, controlling the quantity and quality of microblog content is crucial. For example, Tian et al. (52) found that only 0.37% of posts expressed sleep-related complaints due to the prevalence of advertisements and marketing accounts. Therefore, we carefully screened 923 participants and retained 336 users who had posted a sufficient number of original posts. This ensured a clean high-quality dataset for training the model. Of the 73,735 posts, 3.67% were sleep-related, and 2.15% indicated sleep problems. A unique benefit of our framework is that other researchers can apply our freely available fine-tuned models and datasets to any Chinese post data ^2^. After applying the sleep assessment and personality assessment models to the large microblog dataset (users = 13,753), we found that 12,623 users had made 447,600 posts with sleep-related content (11.3% of all posts), and 12,274 users had made 332,600 posts about sleep problems (8.4% of all posts). These data constitute a rich, high-quality sleep-related microblog dataset.

Our approach is not without limitations. From the perspective of model limitations, the personality assessment models performed modestly for neuroticism and extraversion. One reason for this is that these traits may manifest ambiguously on Chinese social media. For instance, posts related to neuroticism often involve indirect expressions of distress (e.g., sarcasm) rather than explicit emotional disclosure, which aligns with findings by Yuan et al. (42) on cultural differences in personality expression. Extraversion, while theoretically associated with social engagement, could be conflated with performative behaviors online (e.g., frequent but superficial interactions), as noted by Liu and Zhu (46). Similarly, Cutler and Condon (48) reported instability in detecting neuroticism from text, attributing it to the trait’s context-dependent expression. Another interesting, albeit disappointing, finding was that the relationship between openness and sleep quality was inconsistent across the different datasets. Unlike conscientiousness and extraversion, which are descriptive traits, openness is an evaluative trait, which are more susceptible to instability and, consequently, may yield inconsistent results (59). Addressing the stability of openness assessments in future studies could enhance our understanding of its relationship with sleep. Future work could integrate multimodal data (e.g., pictures, interaction patterns) to better capture contextual nuances and improve the stability of assessment (53). Additionally, as our models operate at the post level, the frequency of sleep-related symptoms (e.g., whether a post expresses sleep problems) was not directly incorporated into the deep learning models. Future work could integrate longitudinal data to better capture the frequency and chronicity of symptoms.

Another limitation is that we used a shortened version of the personality questionnaire (i.e., the CBF-PI) for the user survey. Although the CBF-PI has been validated in previous studies, the reduced number of items may have introduced bias in some of the dimensions. Future studies could use the full version and control for other variables that may affect the accuracy of personality assessments. Finally, to explore the relationship between sleep and personality, we used four indicators of sleep characteristics: the number of sleep-related posts, the proportion of sleep-related posts, the number of posts indicating a sleep problem, and the proportion of posts with sleep problems. We found that, without controlling for users’ overall posting activity, these indicators were not significantly correlated with sleep quality. Future research could explore how to assess sleep quality among inactive users (i.e., those who post rarely or not at all). While our models showed significant correlations with PSQI scores, we did not test their ability to directly predict PSQI scores. This limits conclusions about their potential as proxies for standardized sleep assessments. Further validation is needed to assess this predictive capacity in future work.

The study is based on passive data collection from the public social media platform (Sina Weibo), a topic widely discussed in terms of ethical considerations (61, 62). For our study, we took the following ethical precautions. First, we submitted the research for approval from the ethics review board and received approval before proceeding. Second, no private messages were accessed during the research process, and all data (e.g., user IDs and post IDs) were anonymized after preprocessing. Third, the goal of this research is to explore patterns across large populations to derive theoretical insights, rather than applying them to psychological interventions at the individual level. Overall, this study adheres to ethical guidelines, including the APA Guidelines for Telepsychology (63) and the British Psychological Society’s guidelines on internet-mediated research (64). We emphasize that transparent data use disclosures, enhanced data security, and rigorous ethical oversight should be core components of large-scale digital psychological research.

## 5 Conclusions

Our findings demonstrate the reliability of big data-driven computational methods for evaluating sleep characteristics and personality traits. Specifically, we found that: (1) conscientiousness, agreeableness, and extraversion are associated with better sleep quality, while neuroticism is linked to poorer sleep quality. (2) When the model trained on a small survey dataset with expert annotations and questionnaires was applied to a large-scale microblog dataset, the sleep-personality relationships remained consistent across datasets. From a theoretical perspective, our work provides a multifaceted approach that integrates computational methods with psychological research, offering new insights into how big data can inform psychological theory. From the perspective of clinical implications, understanding the relationship between personality traits and sleep characteristics can guide personalized interventions. For example, individuals with high neuroticism may benefit from interventions focused on emotional regulation, such as CBT or mindfulness (65), while those with higher agreeableness, conscientiousness, and extraversion may improve sleep quality with structured routines and social support (66). Future work could explore the underlying causes of sleep problems expressed in microblogs by overcoming data sparsity through multiple data sources and multimodal data approaches. This would facilitate more comprehensive analysis and lead to more precise and effective sleep interventions tailored to individuals’ specific needs.

## Data Availability

The original contributions presented in the study are included in the article/**Supplementary Material**. Further inquiries can be directed to the corresponding author.

## Footnotes

1
https://aishiyan.bnu.edu.cn

2
https://pan.bnu.edu.cn/l/l1wLB0

## Supplementary material

Supplementary Table 1 Correlations between personality traits (CBF-PI scores), PSQI, and model-assessed sleep characteristics in 336 users. O, Openness, C, Conscientiousness, E, Extraversion, A, Agreeableness, N, Neuroticism; PSQI, Pittsburgh Sleep Quality Index; TN, Total Number of Posts; NSR, Number of Sleep-Related Posts; PSR, Proportion of Sleep-Related Posts; NSP, Number of Posts Indicating Sleep Problems; PSP, Proportion of Posts with Sleep Problems; ^*^ *p* <.05, ^**^ *p* <.01.

Supplementary Table 2 Descriptive statistics of users in the large dataset (users = 13,753, posts = 4,864,600). O, Openness; C, Conscientiousness; E, Extraversion; A, Agreeableness; N, Neuroticism; PSQI, Pittsburgh Sleep Quality Index; M, Mean; SD, Standard Deviation; TN, Total Number of Posts; NSR, Number of Sleep-Related Posts; NSP, Number of Posts Indicating Sleep Problems.

Supplementary Table 3 Descriptive statistics of valid surveyed users (users = 336, posts = 73,735). O, Openness; C, Conscientiousness; E, Extraversion; A, Agreeableness; N, Neuroticism; PSQI, Pittsburgh Sleep Quality Index; M, Mean; SD, Standard Deviation; TN, Total Number of Posts; NSR, Number of Sleep-Related Posts; PSR, Proportion of Sleep-Related Posts; NSP, Number of Posts Indicating Sleep Problems; PSP, Proportion of Posts with Sleep Problems; PSQI, Pittsburgh Sleep Quality Index.
