# Supplementary material for: Relationship between personality and sleep: a dual validation study combining empirical and big data-driven approaches
Source: Front Psychiatry. 2025 Jul 17;16:1596269. doi: 10.3389/fpsyt.2025.1596269 (PMC12310568; doi:10.3389/fpsyt.2025.1596269)
Supplement: Supplementary Table 1 — Correlations between personality traits (CBF-PI scores), PSQI, and model-assessed sleep characteristics in 336 users. O, Openness, C, Conscientiousness, E, Extraversion, A, Agreeableness, N, Neuroticism; PSQI, Pittsburgh Sleep Quality Index; TN, Total Number of Posts; NSR, Number of Sleep-Related Posts; PSR, Proportion of Sleep-Related Posts; NSP, Number of Posts Indicating Sleep Problems; PSP, Proportion of Posts with Sleep Problems; * p <.05, ** p <.01. [file Table1.pdf]

SUPPLEMENTAL MATERIALS

Table 1. Correlations between personality traits (CBF-PI scores), PSQI, and model-assessed sleep characteristics in 336 users.

	Gender	Age	Personality self-reported (CBF-PI)				
			O (95% CI)	C (95% CI)	E (95% CI)	A (95% CI)	N (95% CI)
PSQI	0.00	0.02	$-0.15^{**}[-0.26, -0.05]$	$-0.27^{***}[-0.32, -0.11]$	$-0.31^{***}[-0.40, -0.21]$	$-0.29^{***}[-0.39, -0.19]$	$0.50^{***}[0.41, 0.57]$
TN	0.09	0.00	$0.07[-0.04, 0.17]$	$-0.01[-0.12, 0.10]$	$0.06[-0.05, 0.17]$	$0.02[-0.08, 0.13]$	$-0.10[-0.20, 0.01]$
NSR	0.09	0.17^{**}	$-0.12^{*}[-0.22, -0.01]$	$-0.16^{**}[0.27, -0.06]$	$-0.11^{*}[-0.21, 0.00]$	$-0.09[-0.19, 0.02]$	$0.06[-0.05, 0.16]$
PSR	0.05	0.20^{***}	$-0.10[-0.21, -0.01]$	$-0.07[-0.18, 0.04]$	$-0.07[-0.17, 0.04]$	$-0.08[-0.19, 0.03]$	$0.05[-0.06, 0.16]$
NSP	0.12^{*}	0.18^{**}	$-0.14^{*}[-0.24, -0.03]$	$-0.16^{**}[-0.26, -0.06]$	$-0.10[-0.21, 0.01]$	$-0.08[-0.18, 0.03]$	$0.06[-0.05, 0.16]$
PSP	0.07	0.15^{***}	$-0.12^{**}[-0.22, -0.01]$	$-0.04[-0.15, 0.07]$	$-0.06[-0.16, 0.05]$	$-0.06[-0.16, 0.05]$	$0.03[-0.08, 0.14]$

Note. O = Openness, C = Conscientiousness, E = Extraversion, A = Agreeableness, N = Neuroticism; PSQI = Pittsburgh Sleep Quality Index; TN = Total Number of Posts; NSR = Number of Sleep-Related Posts; PSR = Proportion of Sleep-Related Posts; NSP = Number of Posts Indicating Sleep Problems; PSP = Proportion of Posts with Sleep Problems; $^{*}p < .05$, $^{**}p < .01$

Table 2. Descriptive statistics of users in the large dataset (users = 13,753, posts = 4,864,600)

	Gender	O	C	E	A	N	TN	NSR	NSP
M	1.81	10.24	12.55	9.98	12.59	10.53	288.00	32.55	24.18
SD	0.39	0.46	0.50	0.98	0.41	1.32	425.20	60.16	46.67

Note. O = Openness, C = Conscientiousness, E = Extraversion, A = Agreeableness, N = Neuroticism; PSQI = Pittsburgh Sleep Quality Index; M = Mean; SD = Standard Deviation; TN = Total Number of Posts; NSR = Number of Sleep-Related Posts; NSP = Number of Posts Indicating Sleep Problems.

Table 3. Descriptive statistics of valid surveyed users (users = 336, posts = 73,735)

	Age	O	C	E	A	N	PSQI	TN	NSR	PSR	NSP	PSP
M	23.66	10.81	13.34	10.13	13.81	10.33	6.77	219.46	8.08	0.04	4.71	0.02
SD	4.60	3.24	2.54	3.40	2.64	3.28	2.98	329.13	17.89	0.06	10.21	0.04

Note. O = Openness, C = Conscientiousness, E = Extraversion, A = Agreeableness, N = Neuroticism; PSQI = Pittsburgh Sleep Quality Index; M = Mean; SD = Standard Deviation; TN = Total Number of Posts; NSR = Number of Sleep-Related Posts; PSR = Proportion of Sleep-Related Posts; NSP = Number of Posts Indicating Sleep Problems; PSP = Proportion of Posts with Sleep Problems; PSQI = Pittsburgh Sleep Quality Index.
